# Different Supports, Different Effects: Social Support Moderates the Impact of Parenting Stress on the Mental Health of Parents of Individuals with Intellectual and Developmental Disabilities

**DOI:** 10.3390/ejihpe15120248

**Published:** 2025-12-05

**Authors:** Tao Xu, Yiran Zhang

**Affiliations:** Department of Social Work, College of International Education and Social Development, Zhejiang Normal University, Jinhua 321000, China; zhangyiran@zjnu.edu.cn

**Keywords:** parenting stress, social support, mental health of parents of individuals with intellectual disabilities

## Abstract

Parenting stress, social support, and mental health among parents of individuals with intellectual disabilities have garnered significant scholarly attention. Although existing research has extensively elucidated the relationship between parenting stress and mental health, there is limited systematic investigation into the role of social support—particularly regarding which and how social support moderates the impact of parenting stress on the mental health of these parents. Based on data from the 2020 Comprehensive Survey on Caregiving Burden and Public Service Needs of Families with Intellectual Disabilities conducted in Shenzhen, this study examines the relationships among parenting stress, social support, and mental health of Individuals with Intellectual and Developmental Disabilities. The findings reveal that overall, parenting stress exerts a significant negative effect on parental mental health, while social support has a significant positive effect. Further moderation analysis indicates that both subjective support and support utilization partially alleviate the adverse effect of parenting stress on mental health, whereas objective support primarily enhances mental health through a direct pathway. Moreover, differential patterns emerge between parents with and without disabilities: for non-disabled parents, subjective support and support utilization both directly improve mental health and reduce the negative impact of parenting stress. Objective support, however, only contributes directly to mental health and does not show a moderating effect. In contrast, among parents with disabilities, objective support effectively promotes mental health but does not mitigate parenting stress; subjective support and support utilization show neither direct nor buffering effects. These findings enrich the empirical literature on family mental health in the context of intellectual disability and offer practical implications for enhancing family support policies and improving the well-being of affected households.

## 1. Introduction

Individuals with intellectual and developmental disabilities (IDD) refer to those with intellectual disabilities, mental disorders, and those with both (Schalock, 2010). The World Health Organization stated in its 2022 “Global Report on Health Equity for Persons with Disabilities” that currently, 1.3 billion people (16% of the global population) are living with significant disabilities (World Health Organization, 2024). According to statistics from the China Disabled Persons’ Federation in 2023, the total number of registered disabled persons in China is 37.79 million, of whom 3.51 million have intellectual disabilities, 4.43 million have mental disorders, and 2.07 million have multiple disabilities (China Disabled Persons’ Federation, 2024). Individuals with IDD not only face behavioral and communication difficulties but also are often accompanied by physical and mental illnesses such as epilepsy and sleep disorders, requiring continuous medical care and daily support (Kim et al., 2021; Grier et al., 2018). As the primary caregivers, parents of individuals with IDD endure multiple stresses during long-term care (Kumari & Biswas, 2024; Hayes & Watson, 2013). They face not only parenting stress but also challenges from social discrimination, financial burdens, and family role conflicts (Corrigan & Miller, 2004; McStravick & Cousins, 2025). This sustained psychological pressure has been confirmed to significantly impact caregivers’ mental health (Biswas et al., 2015; Shahali et al., 2024; Dunn et al., 2019). Meanwhile, social support, as an important resource for coping with stress, plays a positive role in buffering stress and promoting psychological adaptation (Pandey & Dubey, 2019).

In addition, many studies have been conducted in the context of Western welfare, where formal support systems for families of IDD patients are relatively mature (Villaescusa et al., 2021). In contrast, China is characterized by a rapidly developing but still unbalanced support structure, strong family norms, and a significant amount of caregiving responsibilities borne by parents (Chao, 1994; Lei & Kantor, 2021). These features create a unique social ecology in which the interaction between parenting stress, social support, and mental health may differ from the patterns observed in Western environments. Therefore, exploring the interactive relationship between social support, stress, and mental health of parents of IDD patients in the Chinese context has important theoretical and practical significance for optimizing the support system, improving caregiver well-being, and cultural applicability.

Previous research has primarily explored the mental health issues of parents of individuals with IDD from two directions (Gill & Harris, 1991; Cramm & Nieboer, 2011). On one hand, a large body of research has confirmed that parenting stress is a key factor affecting their mental health. Parents of individuals with IDD who are at a high level of long-term parenting stress tend to experience more negative emotions, such as anxiety, self-blame, and frustration, and face more severe mental health problems (Bitsika & Sharpley, 2004; Miodrag & Hodapp, 2010; AlAnsari & Jahrami, 2018). On the other hand, social support, as a crucial external resource, has a positive impact on mental health. Empirical studies have shown that support from family, friends, and community networks can alleviate psychological stress and improve psychological resilience, thereby helping to improve the mental health status of parents of individuals with IDD (Ben-Zur et al., 2005; Israr & Ahmad, 2019). Previous studies have shown that parenting stress is significantly negatively correlated with the social support received by parents of children with disabilities (Miranda et al., 2019; Fu et al., 2023). From the perspectives of parenting stress and social support, the aforementioned studies have provided a relatively detailed explanation of their relationship with mental health. However, there is still a lack of systematic research on the role of social support in the relationship between parenting stress and mental health, and the exploration of the specific mechanism of whether social support moderates the impact of parenting stress on the mental health of parents of individuals with IDD remains insufficient.

Based on this, this study proposes the following core questions: (1) How does parenting stress affect the mental health of parents of children with intellectual and developmental disabilities? (2) Does social support, and especially how would social support (including subjective support, objective support, and support utilization), mitigate the negative impact of parenting stress on mental health through a moderating effect? Through empirical analysis, this study aims to verify the moderating role of social support and explore the specific mechanisms of different types of support, providing a basis for designing targeted interventions and policy support.

## 2. Theoretical Background and Hypothesis

### 2.1. Parenting Stress and Mental Health

In research on families with disabilities, parenting stress is one of the most widely studied topics (Davis & Carter, 2008; Pisula, 2007). Parenting stress is the negative psychological and physiological reaction that arises when there is a mismatch between a parent’s expectations of the resources needed to meet parenting demands and the available resources within the parent–child relationship system. It typically manifests as negative emotions toward oneself and one’s children (Deater-Deckard, 1998; Walsh-Burke & Marcusen, 1999). Being a parent constitutes a social role that exists both within and outside the family, and this role itself is a source of stress (Alexander & Higgins, 1993). Parents must meet their own needs and those of their children, as well as the demands placed on them by other family members and society. Therefore, for some parents, the parenting stress resulting from an inability to meet these expectations can be continuous or chronic (Lepore, 1997). In addition, parental traits such as temperament, beliefs, and health status can also affect the type and severity of stressors, potentially creating additional stressors during the parenting process (Lepore & Evans, 1996).

The Parent–Child-Relationship Stress theory hypothesizes that the sources of parenting stress are composed of three independent components: stress in the parent domain related to the parent’s own issues, stress in the child domain related to the child’s own characteristics, and parental-child dysfunctional stress related to the degree of conflict in the parent–child relationship (Debra Bendell et al., 1989; Eyberg et al., 1993). The Daily Hassles theory further points out that the most common and impactful stressors for parents and children are those in daily life. The accumulation of these low-level, chronic small-scale stressors may be the most important factor in predicting mental health problems (Kohn, 1996). According to the Stress-Coping Model proposed by Folkman and Lazarus, stress arises from the interaction between an individual and their environment (Folkman & Lazarus, 1985). When an individual’s coping mechanisms are inadequate or unable to meet the demands of the environment, stress is produced. Therefore, parenting stress is not only a reflection of objective pressure but also the result of subjective perception. Loyd and Abidin divided parenting stress into three dimensions: Parent Domain, Child Domain, and Difficult Child/Parent–Child Dysfunctional Interaction (Loyd & Abidin, 1985). Of these, the child’s behavioral problems and functional impairments are the main sources of parenting stress in families with individuals with intellectual and developmental disabilities.

Compared to typical parents, parents of individuals with IDD face higher levels of parenting stress because they must undertake long-term and continuous special care responsibilities (Bouma & Schweitzer, 1990; Dyson, 1997), experience anxiety due to their children’s developmental delays and behavioral problems (Beckman, 1983; Bentovim, 1972; Hastings, 2002), and worry about their children’s future social adaptation and independence (Lecavalier et al., 2006).

Existing research shows that the stress endured by parents of children with IDD is multi-dimensional and long-term, primarily in the following aspects: Caregiver burden, economic stress, emotional stress, social adaptation stress, information and decision-making stress, future uncertainty. Each of these aspects is explained below. Caregiver Burden: The daily lives of children with IDD are highly dependent on their parents, requiring 24 h supervision and support, including feeding, dressing, toileting, education, and rehabilitation training. This high-intensity care leads to physical and mental exhaustion for parents, often resulting in “caregiver burnout,” which is the most direct and common stressor. Research indicates that long-term care can lead to parental health problems, such as chronic fatigue and decreased immune function (Pinquart & Sörensen, 2007). Economic Stress: The high costs of special education, rehabilitation therapy, medical care, and assistive devices for children with IDD place a heavy financial burden on families. Research shows that the economic stress in families with children with IDD is significantly higher than in typical families and can lead to long-term financial hardship (Emerson, 2003). Emotional Stress: Parents often experience complex emotions such as guilt, self-blame, frustration, and anger, and feel fear due to social stigma. The child’s behavioral issues (e.g., self-harm, aggression) further exacerbate the emotional burden (Hastings, 2003). Research indicates that parental emotional stress can lead to anxiety or depressive symptoms (Singer, 2006). Social Adaptation Stress: Parents may face social isolation, misunderstanding, or discrimination due to their child’s special circumstances, and family internal tensions or imbalances in spousal relationships can also arise (Jellett et al., 2015). Research shows that a lack of social support significantly increases parents’ psychological burden (Bromley et al., 2004). Information and Decision-Making Stress: Parents must continuously learn knowledge related to IDD and face a myriad of treatment and educational choices, leading to immense decision-making pressure (Pain, 1999). Information overload and uncertainty can lead to decision fatigue. Future Uncertainty: Worries about the child’s independent life, employment, and social integration in adulthood constitute a long-term psychological burden. Research indicates that parental uncertainty about the future is one of the main sources of stress (Carter et al., 2014).

Long-term parenting stress can alter family relationship structures, leading to changes in parents’ social networks and a loss of a sense of life purpose (Gray, 2002; Kohut, 1966). In a chronic and multi-faceted stressful environment, parents of individuals with IDD are more likely to experience problems such as anxiety, depression, social isolation, and a decline in physical and mental health (Almansour et al., 2013; Hayes & Watson, 2013; Olsson & Hwang, 2001). Consequently, parents of individuals with IDD face higher health risks (Shahali et al., 2024; Smith et al., 2012). Among parents of individuals with IDD, parenting stress often manifests as long-term emotional tension, role confusion, feelings of helplessness in coping, and anxiety about future uncertainties (Boyd, 2002). Some studies point out that mothers experience higher parenting stress than fathers, and thus face more severe mental health issues (Olsson & Hwang, 2001; Wolf et al., 1989). Additionally, parenting stress may vary depending on the degree and type of the child’s disability (Hodapp et al., 1998; Konstantareas & Homatidis, 1989; Seltzer et al., 2004). Siklos and Kerns compared families with different types of disabilities and noted that the stressors and needs differ across these families (Siklos & Kerns, 2006). Ault et al. analyzed families of individuals with IDD in rural areas and found that due to geographical distance and a lack of resources, their family needs could not be met, leading to poor mental health outcomes under the influence of parenting stress (Ault et al., 2021).

Therefore, this study proposes that parenting stress in parents of individuals with IDD will influence their mental health problems, with greater perceived parenting stress being associated with poorer mental health (Hypothesis 1).

### 2.2. Social Support and Mental Health

Social support is widely considered an important psychological and social resource. It is a psychological and material resource provided by social networks that can reduce psychological stress responses, alleviate mental tension, and improve social adaptability (Cohen, 2004; Q. Li, 1998). Research has shown that individuals who receive more social support generally have better physical and mental health (Broadhead et al., 1983). Social support not only helps individuals cope with life’s pressures and crises (Wang, 2004), but also enhances their psychological adaptability and health levels (Leavy, 1983; Mitchell et al., 1982). Although the positive effect of social support on mental health has been widely confirmed (Beckman, 1983; Blazer, 1982; House et al., 1982), its specific mechanisms still require in-depth exploration (Heller et al., 1986). Cohen pointed out that the key to understanding how social support affects mental health lies in distinguishing and analyzing its different types and pathways of action (Cohen, 1988).

House et al. proposed three dimensions for measuring social support: social networks, social relationships, and social support in the narrow sense (House et al., 1988). Social networks view society as a network of nodes and connections, focusing on structural characteristics such as the size, density, and interaction frequency of an individual’s network. Social relationships evaluate the number and type of existing relationships. Social support in the narrow sense evaluates the types and functions of support resources, such as emotional, instrumental, and informational support. Cohen et al. further distinguished social support into structural support (e.g., marital status, number of relationships) and functional support (e.g., emotional support, material assistance) (Cohen & Syme, 1985). The former reflects objective existences like social networks and relationships, while the latter reflects the actual help an individual receives. Accordingly, House and Kahn’s social networks and social relationships can be classified as structural social support, while social support in the narrow sense can be classified as functional social support. Social support influences an individual’s mental health by affecting their emotions, cognition, and behavior (Cohen, 1988).

In the context of mental health, social support helps regulate an individual’s psychological response system. By conveying social expectations, norms, rewards, punishments, and specific aid, it prevents the disorder of psychological functions (Cassel, 1976; Thoits, 1986). Structural social support influences an individual’s health behaviors through a certain degree of social control. For example, social networks help individuals form stronger motivations for healthy behaviors, such as quitting smoking, healthy eating, and regular exercise. The integration of social networks provides individuals with continuous positive emotional support, and meeting social role expectations helps them build a sense of self-worth (Cassel, 1976; Hammer, 1981; Thoits, 1983; Wills et al., 1985), leading to positive self-identity, thereby reducing psychological hopelessness (Thoits, 1986) and strengthening the willingness to self-care (Cohen & Syme, 1985). Functional social support focuses on the direct help an individual receives in a stressful situation. Members of a social network can provide health-related advice, emotional comfort, and material support, thereby helping the individual cope with potential risks and prevent the onset of psychological illness.

From a socio-psychological and constructivist perspective, the self is largely a reflection of how others perceive oneself (Mead, 1934), and the structure of social support reflects an individual’s perception of the external society. The social relationships an individual has within their social network may not in themselves provide actual support, but the individual’s subjective perception of the amount of social support available can largely influence mental health (Lakey et al., 1996). An individual’s belief that others “can provide support when needed” can effectively mitigate their emotional and physiological stress responses to stressful events. Zhu et al. pointed out that perceived support can account for up to 24% of the variance in mental health levels (Zhu et al., 2014). Perceived support strengthens positive beliefs about the self, which in turn has a beneficial effect on health. People who perceive a higher level of social support tend to show fewer emotional problems or mental illnesses (Cohen & Syme, 1985; Rosengren et al., 1993). Conversely, negative expectations of perceived support are likely to overlap with negative self-evaluations, thus inducing emotional distress and mental health problems (Baldwin & Holmes, 1987; Lakey & Cassady, 1990).

Regarding the mechanism by which social support affects mental health, existing studies generally believe there are two main models. The first is the main effect model, which posits that a higher level of social support directly promotes mental health, enhancing well-being and life satisfaction, regardless of whether the individual is in a stressful situation (Cohen & Wills, 1985). The second is the buffering effect model, which emphasizes that social support can act as a moderator when an individual faces a stressful situation, protecting mental health by buffering or weakening the negative impact of stress on it (Wills et al., 1985). Although the understanding of the mechanisms differs, researchers widely agree that social support has a positive impact on mental health. We therefore speculate that social support likely influences mental health through more than one mechanism.

Specifically for parents of individuals with IDD, Sterling constructed a structural equation model that pointed to a bidirectional pathway between mental health and social support (Sterling, 1998). On one hand, a lack of social support significantly increases an individual’s risk of depression, while on the other hand, depressive symptoms can, in turn, weaken an individual’s ability to obtain and perceive support. Researchers generally agree that among parents of individuals with IDD, higher levels of social support are associated with lower levels of depression (Benson, 2012; Gill & Harris, 1991), stress (Tehee et al., 2009), perceived burden (Khanna et al., 2011), and higher levels of mood and health (Ekas et al., 2010; Khanna et al., 2011; Sawyer et al., 2010).

Therefore, this study proposes that there is a significant correlation between social support and the mental health of parents of individuals with IDD, and social support helps reduce mental health problems (Hypothesis 2).

### 2.3. Social Support, Parenting Stress, and Mental Health

Although existing studies have fully revealed the negative relationship between parenting stress and mental health, and have also confirmed the direct positive effect of social support on mental health, the relationship between parenting stress, social support, and mental health is more complex and deserves further analysis.

The discussion on the relationship between social support, parenting stress, and mental health primarily involves two representative theoretical models: the Main Effect Model and the Buffering Effect Model. The Main Effect Model emphasizes that social support generally promotes mental health, regardless of whether the individual is facing a stressful situation (Cohen & Wills, 1985). House et al. pointed out that even in the absence of a stressor, social support can directly promote mental health by providing emotional support, such as a sense of self-esteem and belonging (House et al., 1988). Individuals with higher perceived social support are significantly better off in terms of physical and mental health, whether or not they experience a stressful event (Broadhead et al., 1983). The Buffering Effect Model holds that social support, especially during stressful events, buffers their negative impact on an individual’s mental health by providing informational, emotional, or instrumental support (Cutrona & Russell, 1990). Cassel proposed that social support has a “stress-buffering” effect, where a certain level of social support can mitigate the health risks brought on by stress (Cassel, 1976). Cohen noted that while the Main Effect Model emphasizes the general role of social support and the Buffering Effect Model focuses on its moderating role in stressful environments, both can function simultaneously in different contexts to jointly explain the mechanisms by which social support influences mental health (Cohen, 1988).

Social support can buffer the impact of stress on mental health by changing coping styles and enhancing coping abilities. The key lies in the matching and availability of social support (Cohen, 1988; Cohen & Wills, 1985; Uchino et al., 1996). The Stress-Support Match Hypothesis argues that effective support should match the individual’s actual needs. For example, in times of financial hardship, tangible support is most crucial, whereas emotional support is more important when a loved one passes away (Cutrona & Russell, 1990). The matching of social support and stress can guide people to reduce negative appraisals of stress, thereby mitigating the impact of stress on mental health.

Cohen and Wills proposed that the buffering effect of social support can occur at three stages of a stressful event. Before a stressor occurs, social support can reduce an individual’s risk of exposure to the stressor through a preventive mechanism, for example, by providing effective information, encouraging preventive behaviors, or mobilizing social network resources for protective intervention (Cohen & Wills, 1985; Gottlieb, 1998). During a stressful event, social support can reduce the individual’s subjective perception of stress and lessen the intensity of their emotional response by providing comfort, advice, or direct help (Wills et al., 1985). It can also weaken the negative impact of stress by guiding people to interpret stressful situations less negatively (Cohen & McKay, 1984; Cohen & Hoberman, 1983). After a stressful event, social support can help an individual recover and adapt better, for instance, by enhancing their adherence to a treatment plan and encouraging them to adopt active coping strategies to promote psychological recovery (Wills et al., 1985).

Some studies have also indicated that the buffering effect of social support is highly selective, with the source, type, and satisfaction of support all affecting its buffering capacity (Chisholm et al., 1986; Ganster & Victor, 1988). A mismatch between support and needs may lead to a negative buffering effect, where social support increases the negative impact of stress (Abdel-Halim, 1982; Kaufmann & Beehr, 1986; Winnubst et al., 1982). Specifically, the buffering effect primarily functions through two dimensions: direct support and perceived support. Direct support is the immediate help provided by others when an individual is facing stress, including help with daily life, medical assistance, and financial aid (Berkman, 1985; House, 1981). This support can meet an individual’s practical needs during a state of stress, thereby alleviating the burden of external stressors and maintaining their mental health. Even more important is perceived support, which is the individual’s subjective perception of social support. Perceived support itself is inherently positive and can regulate an individual’s emotions and psychological resilience (Cohen & Syme, 1985; Kessler & McLeod, 1985).

Stress can weaken an individual’s self-esteem, self-efficacy, and sense of social control, which in turn increases the risk of depression and psychosomatic illnesses (Wills et al., 1985). The intervention of social support, especially from interactions and involvement in close relationships, can not only restore an individual’s psychological stability but also enhance their confidence in receiving future help (Foa, 1971). Self-esteem theory and personal control theory (Paulhus & Christie, 1981) also emphasize that an individual’s perception of their own status and sense of control is crucial in stressful situations. Social support helps maintain these key psychological states, thereby promoting positive emotional responses and effective coping behaviors (Reid & Ziegler, 1981).

In the context of families with individuals with IDD, the condition has a profound and lasting impact on the family and its members (X. Li & Zhang, 2018). Consequently, parents of individuals with IDD face greater parenting stress, and their mental health levels and social support are significantly lower than the national norms (Zhang et al., 2016). Gill and Harris conducted a more in-depth exploration of the relationship between mental health, stress, and social support among mothers of children with autism (Gill & Harris, 1991). They proposed that social support, as a predictor of mental health, has a buffering effect that can improve the impact of stress. Parsons et al. built a logistics regression model and found a significant positive correlation between the availability of social support and caregiver mental health, with the effect being more pronounced in high-stress environments (Parsons et al., 2020).

Therefore, this study proposes that social support has a moderating effect on the relationship between parenting stress and the mental health of parents of individuals with IDD, and thus social support can mitigate the negative impact of parenting stress on their mental health (Hypothesis 3).

## 3. Data Sources and Analysis Methods

### 3.1. Data Sources

The data used in this study were collected from the “Comprehensive Survey of Caregiving Burden and Public Service Needs of Families with Individuals with Intellectual and Developmental Disabilities” conducted in Shenzhen in 2020. This survey was part of a larger, nationwide baseline survey aimed at families of individuals with IDD. It aimed to systematically assess the living conditions, caregiver strain, and gaps in public services for these families. In its execution, the survey adopted a community-based convenience sampling approach. The core methodology involved establishing partnerships with well-rooted local organizations, such as parent associations, rehabilitation centers, and special needs schools, using their member families as the primary sampling frame. This was supplemented by snowball sampling, where participating families were asked to refer others. The data collection phase employed a flexible and multi-modal strategy to ensure broad participation: online questionnaires were widely distributed for convenient self-completion, while, simultaneously, trained interviewers or social workers conducted structured face-to-face interviews or provided assisted form-filling for families with limited digital access or to gather more in-depth qualitative data. Ultimately, the professionally cleaned and analyzed data yielded an evidence-based report that comprehensively detailed the families’ real-world challenges and needs, providing a critical foundation for policy advocacy and service improvement. All respondents provided written informed consent prior to data collection. During data processing, cases with missing values were removed in accordance with standard procedures. After data cleaning and preparation, a total of 750 valid cases were retained for the final analyses.

### 3.2. Description of Variables

#### 3.2.1. Dependent Variable

The dependent variable is mental health. Mental health is a multidimensional concept that not only refers to the absence of mental illness but also to a continuous state in which an individual possesses vitality, positive inner experiences, good social adaptability, and is able to effectively utilize their physical and mental potential and positive social functions. This study uses the “Quality of Life Scale” section of the survey questionnaire, which adopted the World Health Organization’s Quality of Life Assessment Scale, short version (The WHOQOL Group, 1998). This scale includes 26 items covering four domains: physical, psychological, social relationships, and environment, to measure the respondents’ quality of life and health status. The psychological domain of the scale consists of 7 items, namely “Are you satisfied with your health?”, “Do you enjoy life?”, “Do you feel your life is meaningful?”, “Can you concentrate?”, “Do you think you look good?”, “Are you satisfied with yourself?”, and “Do you have negative feelings?”. Each item is scored on a 1–5 scale, with a total score ranging from 7 to 35. A lower score indicates a poorer state of mental health.

#### 3.2.2. Independent Variables

##### Parenting Stress

Parenting stress refers to the pressure that parents feel within their parent–child system. This stress often arises from various obstacles that caregivers face due to personal factors, child factors, economic factors, or problems with the support system when fulfilling their role. This study measures the level of parenting stress among parents of children with disabilities using a short form of the Parenting Stress Index (PSI-SF), which was translated and revised by Taiwanese scholar Wen (Wen, 2011). The PSI-SF includes three subscales: Parental Distress (PD), Parent–Child Dysfunctional Interaction (PCDI), and Difficult Child (DC), with a total of 36 items. The questionnaire uses a 5-point Likert scale, with a total score ranging from 36 to 180. A higher score indicates a more severe degree of perceived parenting stress.

##### Social Support

Social support is the influence that an individual receives through social connections that can alleviate psychological stress responses, relieve mental tension, and improve social adaptability. This study uses the Social Support Rating Scale developed by Xiao (Xiao, 1994). This scale has a total of 10 items and includes three dimensions: subjective support, objective support, and support utilization. Subjective support refers to an individual’s emotional experience and satisfaction with being respected, supported, and understood in society, and is closely related to their subjective feelings. Objective support refers to objective, visible, or actual support, including direct material assistance and the existence and participation in social networks and group relationships; this type of support is independent of the individual’s subjective feelings. Support utilization refers to how an individual utilizes the available support. Among the items, there are 12 single-choice matrix questions, each scored from 1 to 4 points. There are also 2 multiple-choice questions: if “no source” is selected, the score is 0; otherwise, the score equals the number of sources selected, with a maximum of 9 points. The sum of the scores for the 10 items is the total social support score, which ranges from 12 to 66. A higher total score indicates more social support received.

#### 3.2.3. Measurement Reliability and Validity

The study used three standardized instruments: the Quality of Life Scale (WHOQOL-BREF, psychological domain), the Parenting Stress Index Short Form (PSI-SF), and the Social Support Rating Scale (SSRS). To ensure the robustness of the analyses, the validity and reliability of each scale were systematically assessed (see Table 1). For reliability, internal consistency was evaluated using Cronbach’s alpha coefficients, with α values above 0.70 considered acceptable. All three scales demonstrated satisfactory internal consistency, with Cronbach’s alpha coefficients ranging from 0.783 to 0.945. Validity was examined using the Kaiser–Meyer–Olkin (KMO) measure and Bartlett’s test of sphericity to assess the appropriateness of factor analysis. KMO values above 0.7 and significant Bartlett’s test results (*p* < 0.001) indicate good construct validity. All three scales met these criteria, supporting their suitability for the current sample. These reliability and validity statistics were calculated based on the present study’s sample of 750 participants.

#### 3.2.4. Control Variables

Prior research has shown that sociodemographic factors are associated with parents’ mental health and may influence levels of parenting stress and social support (Simon, 1995; Miodrag & Hodapp, 2010; Ben-Zur et al., 2005; Blazer, 1982). The control variables in this study include gender, marital status, employment status, health status, and years of education. Gender was converted into a dummy variable, with 1 for female and 0 for male. Marital status was also converted into a dummy variable, with 1 for married (first marriage/remarriage) and 0 for other statuses. Similarly, health status was converted into a dummy variable, with 1 for having a disability and 0 for no disability. Years of education refer to the length of time parents have received formal education, with the standard being: illiterate = 0, primary school = 6, junior high school = 9, high school = 12, associate degree = 15, bachelor’s degree = 16, master’s degree = 18, and doctoral degree = 21.

### 3.3. Analysis Methods

#### 3.3.1. Parameter Testing

To explore differences in mental health levels among groups with different demographic characteristics, this study used an independent samples t-test (for dichotomous variables), one-way analysis of variance (ANOVA) (for categorical variables with multiple levels), and the least significant difference (LSD) method. This analysis helps to reveal the vulnerable situations of specific social groups in terms of mental health.

The formula for the independent samples *t*-test is

$$t=\frac{\bar{X_{1}}-\bar{X_{2}}-(\mu_{1}-\mu_{2})}{\sqrt{\sigma_{12}^{2}}}$$

where the variance is $\sigma_{12}^{2}$, and the mean of the normal distribution is $\mu_{1}-\mu_{2}$.

The formula for one-way ANOVA is

$$x_{ij}=\mu +a_{i}+\varepsilon_{ij}\;\left(i=1,2,\ldots \ldots ,k;j=1,2,\ldots \ldots ,r\right)$$

where the unbiased estimate of is $\hat{\mu }=\bar{x}$, and the unbiased estimate of $a_{i}$ is $\hat{a_{i}}=\bar{x_{i}}-\bar{x}$.

The formula for the LSD method is

$$t=\frac{\left(\bar{x_{i}}-\bar{x_{j}}\right)-\left(\bar{\mu_{i}}-\bar{\mu_{j}}\right)}{\sqrt{MSE(\frac{1}{n_{i}}+\frac{1}{n_{j}})}}$$

where *MSE* is the within-group variance of the observed variable, and $\bar{x_{i}},\bar{x_{j}}$ are the sample means and $\bar{n_{i}},\bar{n_{j}}$ are the sample sizes for the *i*th and *j*th levels, respectively.

#### 3.3.2. Regression Analysis

To further explore the influencing factors of mental health, this study employed a multiple linear regression model. The total mental health score was used as the dependent variable, parenting stress and social support variables as independent variables, and demographic variables as controls. This model can effectively analyze the independent effects of multiple variables on mental health and evaluate their relative importance. The regression equation is as follows:

$$Y=B_{0}+B_{1}X_{1}+B_{2}X_{2}+\ldots +B_{n}X_{n}+\varepsilon$$

where Y is the dependent variable, $B_{0}$ is the constant term, $X_{1}$, $X_{2}$, …, $X_{n}$ are the independent variables, $B_{1}$, $B_{2}$, …, $B_{n}$ are the regression coefficients for each independent variable, and ε is the error term.

#### 3.3.3. Moderating Effect Analysis

To further explore the complex mechanisms of the relationships between variables, this study introduced a moderating effect analysis model to test whether parenting stress moderates the effect of social support on mental health. By adding the interaction term between social support and parenting stress, the significance of the moderating effect can be determined. The expression for the moderation model is as follows:

$$Y=B_{0}+B_{1}X+B_{2}M+B_{3}XM+\varepsilon$$

where X is social support, M is parenting stress, and XM is the interaction term. If $B_{3}$ is significant, it indicates the presence of a moderating effect.

## 4. Results

### 4.1. Descriptive Statistics and Group Comparisons

First, a descriptive statistical analysis was conducted on the parenting stress, social support, and mental health of parents of individuals with intellectual and developmental disabilities (IDD). The results show that the total parenting stress scores for these parents are generally high. An F-test and t-test were then performed on the parents’ scores on the parenting stress, social support, and mental health scales to compare the perceived parenting stress, acquired social support, and mental health status differences they experienced while raising individuals with IDD.

Based on the results in Table 2, the following observations can be made. There were no significant differences in parenting stress, social support, or mental health based on gender and health status (*p* > 0.1). Marital status had a significant impact on social support (*p* < 0.01), with the scores of the married group being generally higher. Employment status had a significant impact on scores across multiple dimensions. The unemployed group faced significantly higher parenting stress than the employed group (*p* < 0.1), while employed parents also scored significantly higher on social support and mental health than the unemployed group (*p* < 0.01). These results indicate that marital status and employment status have a significant impact on the mental health, parenting stress, and social support of parents of individuals with IDD.

### 4.2. Correlation Analysis

Using Pearson correlation analysis, we examined the relationship between parenting stress, social support, and the mental health of parents of individuals with intellectual and developmental disabilities. The results of Table 3 show a significant negative correlation: There was a significant negative correlation between parenting stress and mental health (r = −0.482, *p* < 0.05), indicating that the greater the parenting stress perceived by parents of individuals with IDD, the poorer their mental health. A significant negative correlation existed between parenting stress and social support (r = −0.272, *p* < 0.05), indicating that the less social support parents of individuals with IDD perceive, the greater their perceived parenting stress. A significant positive correlation was found between social support and mental health (r = 0.257, *p* < 0.05), indicating that the higher the social support parents of individuals with IDD perceive, the better their mental health.

### 4.3. Regression Analysis Results

We further used a linear regression analysis to examine the predictive effects of parenting stress and social support on mental health. To distinguish the influences of multiple factors, such as parenting stress, social support, marital status, and years of education on the mental health of parents of individuals with IDD, four models were constructed. Model 1 examined the effects of marital status, employment status, and years of education on mental health. Model 2 examined the effect of social support on mental health. Model 3 examined the effect of parenting stress on mental health. Model 4 examined the combined effects of both parenting stress and social support on mental health.

The results (see Table 4) showed that after including control variables such as gender, marital status, employment status, health status, and years of education in Model 1, the model’s explanatory power (R^2^) was only 0.033. Of the variables, employment status had a significant positive effect on mental health (β = 0.129, *p* < 0.01), and health status had a significant negative effect (β = −0.081, *p* < 0.05). The effects of gender, marital status, and years of education were not significant (*p* > 0.1). In Model 2, with the addition of the social support variable, the model’s explanatory power (R^2^) increased to 0.086. Social support had a significant positive predictive effect on mental health (β = 0.242, *p* < 0.01), while the effects of employment status (β = 0.093, *p* < 0.05), health status (β = −0.076, *p* < 0.05) remained significant.

In Model 3, with the introduction of the parenting stress variable, the model’s explanatory power (R^2^) significantly increased to 0.249. Parenting stress had the strongest negative predictive effect on mental health (β = −0.475, *p* < 0.01). In the final Model 4, where both social support and parenting stress were included, the model’s explanatory power (R^2^) further increased to 0.264. Parenting stress remained the most significant negative predictor (β = −0.443, *p* < 0.01), while social support still had a positive effect on mental health (β = 0.128, *p* < 0.01). Both were significant predictors based on the control variables. Hypothesis 1 and Hypothesis 2 were both confirmed.

The results above indicate that the negative effect of parenting stress on mental health is significant, and although the effect of social support weakened after including parenting stress in the model, it still plays a positive role in mental health. This suggests that social support may, to some extent, buffer the negative impact of parenting stress on mental health. Therefore, this study will further test the moderating effect of social support on the relationship between parenting stress and mental health to gain a deeper understanding of their interaction mechanism.

### 4.4. Moderation Effect Analysis Results

Given that social support is a multidimensional concept and different types of support may play distinct roles in the buffering mechanism, this study disaggregated social support into three dimensions for analysis: subjective support, objective support, and support utilization. To explore the specific moderating effects of these three types of support on the relationship between parenting stress and mental health, the scores for each type of support were centered and then included as moderating variables in the regression equation for testing.

**Figure 1:**
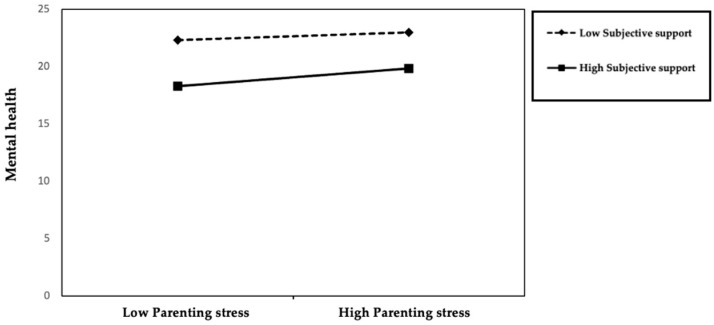
The moderating effect of Subjective Support.

Based on the moderation analysis results (see Table 5), subjective support showed a degree of moderating effect on the relationship between parenting stress and mental health. In model 5, parenting stress significantly negatively predicted mental health (β = −0.446, *p* < 0.01), while subjective support significantly positively predicted mental health (β = 0.110, *p* < 0.01). This indicates that the higher the level of subjective support parents feel, the better their mental health, and the higher the parenting stress, the poorer their mental health. The model’s explanatory power was R^2^ = 0.260, suggesting that both variables can, to some extent, explain the changes in mental health levels. In Model 6, after adding the interaction term between parenting stress and subjective support, the main effects of parenting stress (β = −0.445, *p* < 0.01) and subjective support (β = 0.123, *p* < 0.01) remained significant. Additionally, the interaction term reached a marginal significance level (β = 0.063, *p* < 0.1), and the model’s explanatory power increased to R^2^ = 0.264, with a change in R^2^ of 0.004. This result suggests that subjective support has a certain buffering effect on the relationship between parenting stress and mental health. Specifically, in situations of high parenting stress, subjective support can partially weaken the negative impact of stress on mental health, thereby playing a protective moderating role. Subjective support not only directly and positively influences mental health but also moderates the negative impact of parenting stress, meaning that when parents face high parenting stress, a high subjective perception of available support can mitigate the declining trend of their mental health (Figure 1). Subjective support, therefore, plays a dual positive role in maintaining the mental health of parents of individuals with IDD.

In model 7, parenting stress significantly negatively predicted mental health (β = −0.459, *p* < 0.01), and objective support significantly positively predicted mental health (β = 0.093, *p* < 0.01). This indicates that even after controlling for education, marital status, and employment status, parenting stress and objective support can still significantly explain the variance in mental health levels. The positive main effect of objective support suggests that tangible support resources obtained from social networks have a positive effect on improving parental mental health. However, after adding the interaction term between parenting stress and objective support in the second step of the regression, the main effects of parenting stress (β = −0.464, *p* < 0.01) and objective support (β = 0.095, *p* < 0.01) remained significant, but the interaction term did not reach a significant level (β = 0.052, *p* > 0.1). The model’s explanatory power only slightly increased to R^2^ = 0.260, with a change in R^2^ of 0.003. This result indicates that, despite its direct positive effect on mental health, objective support does not play a significant buffering role in moderating the relationship between parenting stress and mental health.

**Figure 2:**
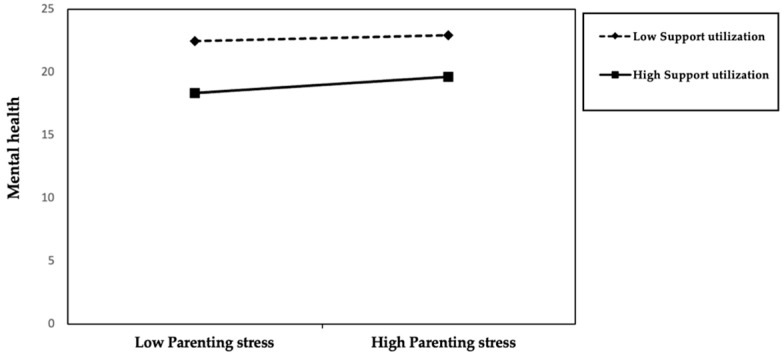
The moderating effect of Support Utilization.

Support utilization also showed a degree of moderating effect on the relationship between parenting stress and mental health. In model 9, parenting stress significantly negatively predicted mental health (β = −0.455, *p* < 0.01), while support utilization significantly positively predicted mental health (β = 0.102, *p* < 0.01). This indicates that after controlling for variables like education, marital status, and employment status, the higher an individual’s support utilization, the better their mental health, and the greater the parenting stress, the poorer their mental health. In model 10, after introducing the interaction term between parenting stress and support utilization, the main effects of parenting stress (β = −0.457, *p* < 0.01) and support utilization (β = 0.106, *p* < 0.01) remained significant. The interaction term (β = 0.056, *p* < 0.1) was also significant, and the model’s explanatory power increased to R^2^ = 0.262, with a change in R^2^ of 0.003. This result suggests that support utilization may have a certain buffering effect between parenting stress and mental health (Figure 2). That is, in situations of high parenting stress, if parents can more actively and consciously use or mobilize existing social resources, the extent to which their mental health is negatively affected may be relatively lower. Therefore, support utilization not only improves mental health through a direct path but may also, to some extent, mitigate the negative impact of parenting stress on mental health. Hypothesis 3 was almost confirmed.

Considering that the effects of social support and parenting stress on mental health may differ between parents with disabilities and those without, we constructed separate models for each group. The results (shown in Table 6) indicate that for parents without disabilities (Models 11–16), parenting stress had a significant negative effect on mental health, suggesting that higher parenting stress is associated with lower mental health levels. Among the three types of social support, subjective support (β = 0.107, *p* < 0.01), objective support (β = 0.084, *p* < 0.1), and support utilization (β = 0.115, *p* < 0.01) all showed significant positive effects on mental health, indicating that all three dimensions of social support effectively enhance mental health in parents without disabilities. Additionally, the interaction terms of subjective support (β = 0.070, *p* < 0.1) and support utilization (β = 0.059, *p* < 0.1) with parenting stress were both significant, supporting the buffering hypothesis of social support. In contrast, the interaction term of objective support (β = 0.049, *p* > 0.1) was not significant, which is consistent with the earlier analysis.

For parents with disabilities (Models 17–22), the negative effect of parenting stress remained significant, but the role of social support was relatively weaker. Neither the main effects nor the interaction terms of subjective support and support utilization reached significance, with only the main effect of objective support being significant (β = -0.376, *p* < 0.1). This implies that for parents with disabilities, objective social support plays a more critical role.

A comparison between the two groups revealed that the negative effect of parenting stress on mental health was significant in both groups, indicating that, regardless of disability status, higher parenting stress significantly reduces mental health levels (Table 7).

However, there were notable differences in the promoting and buffering effects of social support between parents with and without disabilities. For parents without disabilities, both subjective support and support utilization not only directly promoted mental health but also mitigated the negative impact of parenting stress. In contrast, objective support primarily contributed to mental health without demonstrating a buffering effect. For parents with disabilities, objective support effectively promoted their mental health but did not alleviate parenting stress. Subjective support and support utilization, on the other hand, showed neither direct promoting effects nor indirect buffering effects.

## 5. Discussion and Conclusions

### 5.1. Discussion

This study investigates the impact of social support on the mental health of parents of individuals with IDD by analyzing comprehensive survey data on family caregiving burdens and public service needs. Based on the results of the data analysis, this paper identifies new pathways through which social support influences the mental health of these parents.

Firstly, the study finds that parenting stress significantly increases mental health problems among parents of individuals with IDD. Regression analysis shows that higher parenting stress is associated with poorer mental health outcomes. This finding aligns closely with existing research, which consistently indicates that parents of individuals with IDD face elevated parenting stress due to factors such as long-term caregiving burdens, lack of social support, and strained family resources. These factors contribute to persistent and significant psychological stress. When this stress exceeds an individual’s coping capacity, it is likely to trigger mental health issues such as depression and anxiety. For instance, Zhang et al. found that low levels of social support are a key factor contributing to poor mental health among family members of individuals with depression (Zhang et al., 2016). Similarly, Australian research shows that over 80% of parents of individuals with IDD report feeling “overwhelmed by stress,” exhibiting anxiety and depression levels above the norm (Bitsika et al., 2013). Studies from the United States, the United Kingdom, and Sweden also confirm that parenting stress exacerbates mental health problems for these parents (Hastings, 2002; Kohut, 1966; Olsson & Hwang, 2001).

Secondly, this study verifies the positive effect of social support on mental health. Social support is significantly positively correlated with the mental health of parents of individuals with IDD, supporting the main effect model’s view that social support has a broadly positive impact. The results indicate that subjective social support, objective social support, and support utilization directly promote parents’ mental health, mitigating negative emotions in stressful situations and reducing the adverse impact of parenting stress on mental health. This finding is consistent with prior research. For example, Weiss found that mothers’ perceptions of social support were significantly associated with reduced depressive symptoms in a study of mothers of children with autism (Weiss, 2002). Similarly, Ekas noted that perceived support was directly or indirectly related to parents’ distress and well-being (Ekas et al., 2010). However, this study also reveals limitations in the social support available to families of individuals with IDD. The overall support scores for the sample were generally low, particularly for support utilization, with a mean score of only 6.84, indicating that these families struggle to effectively mobilize social resources in daily life.

Thirdly, this study further validates the moderating role of social support in the relationship between parenting stress and mental health. Existing research has confirmed that social support can alleviate mental health problems when parents face excessive parenting stress (Israr & Ahmad, 2019; Kessler & McLeod, 1985; Winnubst et al., 1982). However, prior studies have not clarified how social support functions in stressful situations to improve mental health. By disaggregating social support into subjective support, objective support, and support utilization, this study finds that the moderating mechanisms are not uniform. Subjective support and support utilization exhibit a moderating effect, with subjective support showing a particularly significant impact. This suggests that individuals’ subjective perceptions of support and their ability to utilize it play a critical role in mitigating the effects of stress.

The mechanisms of subjective support may operate in two ways. On the one hand, subjective support helps alleviate parents’ emotional distress. Existing research indicates that a key pathway of social support is reducing negative emotions and enhancing emotional regulation (Wills et al., 1985). This study finds that mental health scores are significantly better among groups with higher subjective support, possibly because, in high-pressure caregiving environments, subjective support fosters a belief that others understand and can provide help, reducing feelings of helplessness. On the other hand, subjective support enhances parents’ sense of self-worth and control. Parents of individuals with IDD often feel marginalized due to role-related stress and social isolation, and emotional interactions and support to help maintain their self-esteem and confidence. Thus, in these families, subjective support may play a deeper role in sustaining long-term mental health compared to objective support. In contrast, while objective support has a direct positive effect on mental health, its moderating effect is not significant. This suggests that the mechanisms of different types of social support vary: compared to merely possessing external resources, individuals’ subjective perceptions and proactive support utilization are more critical in alleviating the psychological burden caused by stress. Simply providing resources or services may not sufficiently address psychological distress.

Fourthly, the study finds differences in the promoting and buffering effects of social support between parents with disabilities and those without. For non-disabled parents, subjective support and support utilization not only directly promote mental health but also buffer the negative impact of parenting stress on mental health. Objective support primarily promotes mental health but lacks a buffering effect. For parents with disabilities, objective support effectively promotes mental health but does not buffer the impact of parenting stress, while subjective support and support utilization neither directly promote mental health nor provide indirect buffering effects. These findings refine the understanding of social support’s mechanisms across different groups, addressing gaps in prior research regarding variations in these mechanisms and enabling a more precise exploration of social support’s pathways.

These results align with both the main effect and buffering effect models of social support. Overall support contributes directly to better mental health, consistent with the main effect model, while the differentiated impact of subjective and objective support under varying family conditions illustrates the buffering effect, showing how support moderates the relationship between caregiving stress and parental mental health. This conceptual integration highlights the dual role of social support, informing targeted interventions that address both general well-being and stress-specific needs.

These findings provide clear directions for improving the mental health of parents of individuals with IDD at the policy level. First of all, mental health services should be integrated into support systems for special-needs families. Current support systems for children with IDD often focus on the children themselves, overlooking the long-term caregiving burden on parents, which can lead to accumulated negative emotions and increased risks of anxiety and depression. Professionals should proactively identify parents at risk through regular mental health assessments and tailor interventions to their specific needs. Policymakers should allocate funding to ensure these services are accessible and affordable. Community services, disability organization programs, and public health systems should incorporate mental health screenings and interventions for these parents. Furthermore, a systematic social support network for families of individuals with IDD should be established. Through collaboration between government and social organizations, multi-level support services—such as parent support groups, community companionship services, mental health hotlines, and educational and legal consultations—can enhance parents’ ability to identify, mobilize, and utilize social resources. Family support programs should offer structured training for parents on coping strategies, stress management, and navigating available social resources, ensuring that knowledge and practical skills are effectively transferred. Finally, the value of social support lies not only in direct assistance but also in its buffering effect on mental health in high-pressure situations. Interventions should shift from merely providing resources to facilitating their effective use, emphasizing proactive emotional care and psychological empowerment for parents. Mechanisms such as respite care and substitute caregiving should be established to provide parents with periodic breaks, helping maintain psychological resilience, reducing psychological exhaustion from long-term caregiving, and fundamentally mitigating the negative impact of stress on mental health. Policymakers and program designers should prioritize developing policies and programs that enable equitable access to these mechanisms, especially for families in under-resourced communities. Notably, for typical families with IDD, prioritizing subjective social support is key, while for families with disabled parents, increasing objective social support is more effective. Implementing these differentiated approaches ensures that interventions are both targeted and evidence-based, maximizing positive outcomes for parents’ mental health.

Despite achieving expected results, this study has limitations due to objective constraints. First, although the data were systematically collected multi-level data from parents of individuals with IDD, the study relies on self-report data, which may introduce reporting biases. Second, it relies on cross-sectional data, which cannot capture the dynamic and fluctuating nature of parents’ mental health, necessitating longitudinal data for future analysis. Third, although employment status was included as a control variable in the analytical models, the study was unable to further examine the underlying differences in income levels and the associated economic resources that might influence parental stress and mental health. Future research should incorporate more comprehensive economic measures to better elucidate how socioeconomic conditions affect the mental health of parents of individuals with intellectual disabilities. Moreover, the sample primarily consists of parents from Shenzhen, a city with advanced economic development and robust social security systems. Families in such areas often have better access to social support and policy coverage than those in less developed regions, so the data may not fully represent the situation nationwide. Additionally, while comparisons were made with Western studies, China’s unique family structures, cultural values, and public service systems—such as a tendency for parents to internalize responsibility and suppress stress—may amplify the negative effects of parenting stress on mental health and influence the mobilization and acceptance of social support. Thus, whether the moderating pathways and psychological impact mechanisms identified in this study apply to other cultural contexts requires further cross-cultural research.

### 5.2. Conclusions

This study concludes that parenting stress in parents of individuals with IDD significantly increases their mental health problems; and underscores the critical role of social support in mitigating the mental health challenges faced by parents of individuals with IDD, revealing distinct pathways through which subjective support, objective support, and support utilization influence psychological well-being. The findings highlight that subjective support and its utilization are particularly effective in buffering the negative impact of parenting stress, especially for non-disabled parents, while objective support plays a more significant role for parents with disabilities. These insights emphasize the need for tailored interventions that prioritize psychological empowerment and resource utilization over mere provision of services. It also recommends that future policies should integrate mental health screenings, respite care, and multi-level support networks to bolster psychological resilience in these families, with particular attention to cultural and individual differences in support needs. In addition to its relevance within the Chinese context, this study contributes to the international literature on families of individuals with intellectual and developmental disabilities. The findings regarding the main and buffering effects of support provide insights applicable across cultural contexts, offering guidance for interventions and policy design in other countries supporting families of individuals with IDD.

## Figures and Tables

**Table 1:** Reliability and Validity Test Results.

Dimension	Reliability	Validity		Items
	Cronbach’s Alpha	KMO & Bartlett Test	Sig	
Quality of Life Scale (Psychological Domain)	0.845	0.847	0.000	7
Parenting Stress Index (Short Form)	0.945	0.952	0.000	36
Social Support Rating Scale	0.783	0.812	0.000	10

**Table 2:** Descriptive Statistics and Difference Comparison of Parenting Stress, Social Support, and Mental Health among Parents of Individuals with Intellectual Disabilities (M ± SD).

		Parenting Distress	Parent–Child Dysfunctional Interaction Difficult	Difficult Child	Total Parenting Stress	Subjective Support	Objective Support	Support Utilization	Total Social Support	Mental Health Score
Total score		42.04 ± 9.64	33.46 ± 9.04	33.96 ± 8.78	109.47 ± 23.76	8.09 ± 3.09	18.97 ± 4.83	6.84 ± 2.07	33.91 ± 8.14	20.77 ± 4.03
Gender	male	41.28 ± 9.86	34.34 ± 9.53	34.66 ± 9.22	110.29 ± 25.25	8.26 ± 2.99	19.26 ± 4.71	6.69 ± 1.99	34.23 ± 7.87	21.09 ± 3.95
	female	42.44 ± 9.50	33.00 ± 8.75	33.60 ± 8.53	109.04 ± 22.96	8.01 ± 3.14	18.81 ± 4.89	6.92 ± 2.11	33.74 ± 8.27	20.60 ± 4.06
	t	−1.56	1.93	1.57	0.68	1.08	1.21	−1.43	0.76	1.57
Marital status	married	42.08 ± 9.53	33.56 ± 8.94	34.02 ± 8.66	109.66 ± 23.33	8.19 ± 3.11	19.29 ± 4.71	6.84 ± 2.07	34.33 ± 8.05	20.76 ± 4.03
	others	41.66 ± 10.79	32.38 ± 10.05	33.30 ± 10.08	107.35 ± 28.22	6.98 ± 2.58	15.41 ± 4.77	6.87 ± 2.08	29.27 ± 7.76	20.87 ± 4.11
	t	−0.32	−0.97	−0.61	−0.73	−2.97 **	−6.18 ***	0.11	−4.75 ***	0.19
Employment status	employed	43.63 ± 9.05	33.73 ± 8.70	33.93 ± 8.42	111.30 ± 22.37	7.51 ± 2.90	18.47 ± 4.82	6.66 ± 2.10	32.65 ± 7.88	20.23 ± 3.83
	unemployed	40.05 ± 9.99	33.12 ± 9.45	34.00 ± 9.23	107.17 ± 25.24	8.82 ± 3.17	19.59 ± 4.77	7.07 ± 2.02	35.49 ± 8.19	21.45 ± 4.18
	t	5.15 ***	0.92	−0.11	2.37 *	−5.83 ***	−3.15 **	−2.70 **	−4.81 ***	−4.13 ***
Health status	Without Disability	41.96 ± 9.68	33.43 ± 9.03	33.93 ± 8.84	109.34 ± 23.86	8.11 ± 3.10	19.02 ± 4.80	6.83 ± 2.07	33.97 ± 8.10	20.84 ± 4.03
	With Disability	43.75 ± 8.59	34.00 ± 9.31	34.60 ± 7.50	112.36 ± 21.46	7.75 ± 2.79	17.90 ± 5.47	6.96 ± 2.22	32.63 ± 8.88	19.21 ± 3.81
	t	−1.94	−0.34	−0.43	−0.71	0.64	1.29	−0.35	0.92	2.28
Years of education		Mean		Std		Min		max		
		12.11		4.37		0		21		

* *p* < 0.1, ** *p* < 0.05, *** *p* < 0.01.

**Table 3:** Correlation Matrix.

	M ± SD	Parenting Stress	Social Support	Mental Health
Parenting Stress	109.47 ± 23.76	1		
Social support	33.91 ± 8.14	−0.272 **	1	
Mental health	20.77 ± 4.03	−0.482 **	0.257 **	1

* *p* < 0.1, ** *p* < 0.05, *** *p* < 0.01.

**Table 4:** Results of Linear Regression Analysis.

	Model 1	Model 2	Model 3	Model 4
Gender	0.012	−0.006	−0.022	−0.030
	(0.345)	(0.336)	(0.304)	(0.302)
Marital status	−0.013	−0.056	−0.002	−0.025
	(0.532)	(0.526)	(0.469)	(0.474)
Employment status	0.129 ***	0.093 **	0.103 ***	0.085 **
	(0.350)	(0.343)	(0.309)	(0.308)
Health status	−0.081 **	−0.076 **	−0.068 **	−0.066 **
	(0.712)	(0.693)	(0.628)	(0.623)
Years of education	−0.064	−0.028	−0.014	−0.028
	(0.036)	(0.035)	(0.032)	(0.032)
Social support		0.242 ***		0.128 ***
		(0.018)		(0.017)
Parenting stress			−0.475 ***	−0.443 ***
			(0.005)	(0.006)
Constant	19.772	16.916	29.601	27.440
N	750	750	750	750
Adj-R^2^	0.033	0.086	0.249	0.264

* *p* < 0.1, ** *p* < 0.05, *** *p* < 0.01.

**Table 5:** Results of Moderation Effect Analysis.

	Objective Support		Subjective Support		Support Utilization	
	Model 5	Model 6	Model 7	Model 8	Model 9	Model 10
Gender	−0.028	−0.026	−0.024	−0.020	−0.032	−0.032
	(0.303)	(0.302)	(0.303)	(0.303)	(0.304)	(0.303)
Marital status	−0.015	−0.019	−0.023	−0.025	−0.003	−0.004
	(0.470)	(0.470)	(0.480)	(0.480)	(0.467)	(0.466)
Employment status	0.082 **	0.081 **	0.096 **	0.095 **	0.095 **	0.093 **
	(0.311)	(0.310)	(0.308)	(0.307)	(0.307)	(0.307)
Health status	−0.067 **	−0.071 **	−0.066 **	−0.067 **	−0.070 **	−0.070 **
	(0.624)	(0.624)	(0.625)	(0.625)	(0.625)	(0.624)
Education level	−0.021	−0.020	−0.023	−0.021	−0.027	−0.029
	(0.032)	(0.032)	(0.032)	(0.032)	(0.032)	(0.032)
Parenting stress	−0.446 ***	−0.445 ***	−0.459 ***	−0.464 ***	−0.455 ***	−0.457 ***
	(0.006)	(0.006)	(0.006)	(0.006)	(0.006)	(0.006)
Subjective support	0.110 ***	0.123 ***				
	(0.044)	(0.045)				
Parenting stress × Subjective support		0.063 *				
		(0.002)				
Objective Support			0.093 ***	0.095 ***		
			(0.028)	(0.028)		
Parenting stress × Objective support				0.052		
				(0.001)		
Support Utilization					0.102 ***	0.106 ***
					(0.064)	(0.064)
Parenting stress × Support Utilization						0.056 *
						(0.002)
N	750	750	750	750	750	750
Adj-R^2^	0.260	0.264	0.257	0.260	0.259	0.262
ΔAdj-R^2^		+0.004		+0.003		+0.003

* *p* < 0.1, ** *p* < 0.05, *** *p* < 0.01.

**Table 6:** Results of Between-Group Comparison Analysis.

	Parents Without Disability						Parents with Disability					
	Objective Support		Subjective Support		Support Utilization		Objective Support		Subjective Support		Support Utilization	
	Model 11	Model 12	Model 13	Model 14	Model 15	Model 16	Model 17	Model 18	Model 19	Model 20	Model 21	Model 22
Sex	−0.023	−0.020	−0.018	−0.015	−0.028	−0.027	−0.302 *	−0.254	−0.258	−0.252	−0.254	−0.266
	(0.311)	(0.311)	(0.311)	(0.312)	(0.311)	(0.311)	(1.609)	(1.587)	(1.530)	(1.561)	(1.704)	(1.770)
Marital status	−0.014	−0.018	−0.022	−0.024	−0.002	−0.003	−0.059	−0.058	−0.015	−0.011	0.045	0.043
	(0.489)	(0.489)	(0.501)	(0.501)	(0.486)	(0.485)	(1.849)	(1.796)	(1.672)	(1.703)	(1.859)	(1.893)
Employment status	0.072 *	0.071 *	0.086 *	0.085 *	0.083 *	0.082	0.397 *	0.487 *	0.453 *	0.449 *	0.389 *	0.383 *
	(0.321)	(0.321)	(0.318)	(0.318)	(0.317)	(0.316)	(1.490)	(1.511)	(1.443)	(1.468)	(1.530)	(1.564)
Years of education	−0.014	−0.013	−0.014	−0.013	−0.022	−0.024	−0.194	−0.247	−0.314	−0.290	−0.148	−0.134
	(0.032)	(0.032)	(0.033)	(0.033)	(0.033)	(0.033)	(0.169)	(0.167)	(0.174)	(0.186)	(0.175)	(0.183)
Parenting stress (PS)	−0.456 ***	−0.454 ***	−0.470 ***	−0.473 ***	−0.462 ***	−0.465 ***	−0.370 *	−0.345 *	−0.376 *	−0.391 *	−0.372 *	−0.370 *
	(0.006)	(0.006)	(0.006)	(0.006)	(0.006)	(0.006)	(0.031)	(0.031)	(0.030)	(0.031)	(0.033)	(0.033)
Subjective support (SS)	0.107 ***	0.123 ***					0.227	0.324 *				
	(0.045)	(0.046)					(0.246)	(0.253)				
PE × SS		0.070 *						−0.295				
		(0.002)						(0.012)				
Objective support (OS)			0.084 *	0.086 *					−0.376 *	0.354 *		
			(0.029)	(0.029)					(0.030)	(0.124)		
PE × OS				0.049						0.070		
				(0.001)						(0.007)		
Support utilization					0.115 ***	0.119 ***					−0.083	−0.090
					(0.065)	(0.065)					(0.327)	(0.335)
PE × SU						0.059 *						0.055
						(0.002)						(0.015)
N	750	750	750	750	750	750	750	750	750	750	750	750
Adj-R^2^	0.261	0.266	0.258	0.260	0.264	0.267	0.275	0.342	0.338	0.342	0.237	0.239

* *p* < 0.1, ** *p* < 0.05, *** *p* < 0.01.

**Table 7:** Overview of Different Model Results (Models 11–22).

Group	Model	Variables	Significant	Adj-R^2^	ΔAdj-R^2^
Parents without disability	Model 11	Subjective support	Yes	0.261	-
	Model 12	Subjective support	Yes	0.266	+0.005
		Interaction term:	Yes		
	Model 13	Objective support	Yes	0.258	-
	Model 14	Objective support	Yes	0.260	+0.002
		Interaction term:	No		
	Model 15	Support utilization	Yes	0.264	-
	Model 16	Support utilization	Yes	0.267	+0.003
		Interaction term:	Yes		
Parents with disability	Model 17	Subjective support	No	0.275	-
	Model 18	Subjective support	Yes	0.342	+0.067
		Interaction term:	No		
	Model 19	Objective support	Yes	0.338	-
	Model 20	Objective support	Yes	0.342	+0.004
		Interaction term:	No		
	Model 21	Support utilization	No	0.237	-
	Model 22	Support utilization	No	0.239	+0.002
		Interaction term:	No		

ΔAdj-R^2^ represents the change in adjusted R^2^ after adding the interaction term.

## Data Availability

The data presented in this study are available upon request from the corresponding author.
